# Inpatient Telehealth and Coronavirus Disease 2019 Outcomes: Experiences in Alabama

**DOI:** 10.1089/tmr.2021.0004

**Published:** 2021-05-18

**Authors:** Kierstin Cates Kennedy, Kristine R. Hearld, Brian May, Allyson G. Hall, Sue S. Feldman, Kyndal McKnight, Abigayle Kraus, Wendy Feng, William Opoku-Agyeman

**Affiliations:** ^1^ UAB Hospital | Hospital Medicine, The University of Alabama at Birmingham, Birmingham, Alabama, USA.; ^2^ Department of Health Services Administration, School of Health Professions, The University of Alabama at Birmingham, Birmingham, Alabama, USA.; ^3^ School of Medicine, The University of Alabama at Birmingham, Birmingham, Alabama, USA.; ^4^ School of Health and Applied Human Sciences, University of North Carolina at Wilmington, Wilmington, North Carolina, USA.

**Keywords:** COVID-19, Hospital Medicine, telehealth, telemedicine

## Abstract

**Background:** During the early months of the coronavirus disease 2019 (COVID-19) pandemic, hospitals were concerned about preserving personal protective equipment. UAB Hospital Medicine designed a strategy to outfit acute care patient rooms on a COVID-19 unit with telemedicine technology to allow for remote clinician rounding.

**Objective:** To describe one hospital's experience with inpatient telehealth and compare outcomes between patients with and without inpatient telehealth visits.

**Design and Methods:** Retrospective chart review of patients admitted to UAB Hospital Medicine with COVID-19 between March 16, 2020 and April 24, 2020. Logistic and negative binomial regression models were used to examine the relationship between telehealth visits and the likelihood of a subsequent transfer to the intensive care unit (ICU), ventilation, and number of ICU days. Clinician interviews provided additional insight into the telehealth implementation.

**Findings:** One-quarter of the patients received a telehealth visit. Half were admitted to the ICU, and one-third received ventilation. Regression models did not identify statistically significant differences in transfer to the ICU, number of ICU days, and ventilation between patients with and without telehealth visits. Older age and increased respiratory rate were associated with higher odds of ICU admission. Patients with a cough were associated with lower odds of ventilation and fewer ICU days.

**Discussion:** Implementation challenges included difficulties associated with assisting patients with operating the tablets. However, clinicians noted that there was a great benefit to patients being able to see an unmasked physician. Furthermore, the telehealth program proved to be a viable strategy for connecting patients in isolation with their families. Findings can inform the future development of inpatient telemedicine strategies.

## Background

University Hospital is a 1157-bed academic medical center located in Birmingham, Alabama, in the United States. In March 2020, as the threat of severe acute respiratory syndrome coronavirus 2 (coronavirus disease 2019 [COVID-19]) became a reality in Alabama, UAB Hospital Medicine committed to providing inpatient care for all non-ICU COVID patients admitted to the institution by dedicating two Hospital Medicine nursing units to cohort the COVID-19 patients.

During the early months of the COVID-19 pandemic (March–June 2020), personal protective equipment (PPE) was in short supply at University Hospital and elsewhere. To be more efficient in using PPE, University Hospital piloted a telehealth strategy for hospital units that were treating COVID-19 confirmed and suspected cases. An inpatient telehealth strategy is useful because it can reduce the number of people entering a patient's room, reducing opportunities for exposure, and preserving stock of PPE.^1–4^ During the pandemic, other health systems also reported using inpatient telemedicine to provide dermatology,^5,6^ palliative care,^7^ and diabetes-related consults,^8^ as well as to support inpatient care in general.^9^ These reports, which are mostly descriptive, concluded that inpatient telemedicine resulted in reduced use of PPE.^6–8^ Interestingly, the study on diabetes-related consults reports no changes in glycemic control among patients with diabetes.^8^ However, telemedicine may have limits especially with accessibility, communication, and hearing limitation or when a physical examination is needed.^7^

University Hospital partnered with UAB eMedicine to adapt current outpatient telemedicine technology (American Well™) for use in the inpatient setting. Working with nursing staff, Hospital Medicine clinical care coordinators (CCCs), hospitalists, and subspecialist physicians designed a strategy to outfit acute care patient rooms on a medical-surgical COVID-19 unit with telemedicine technology (electronic tablets or telemedicine carts) that would allow for remote rounding by physicians and advanced practice providers (clinicians). Nursing staff were tasked with ensuring that tablets were in the room and within easy reach of the patient. Before implementation, nursing and medical staff viewed a training video that provided instruction on how to download and use the telemedicine applications.

University Hospital piloted two versions of inpatient telehealth. Version 1 involved electronic tablets placed in each patient's room on the nightstand or overbed table. Version 2 was a telemedicine cart that included a monitor and a keyboard. When the clinician indicated rounds were starting, the clinician notified a nurse who functioned as the CCC. The CCC called the patient in the room and guided the clinicians and patient connections through the technology. The patient and the clinicians were invited into a virtual room. Using an e-mail or short message system text message link, family members could also be invited into the virtual room. The CCC exited the virtual room once the clinician, patient, and the patient's family were connected. All encounters continued remotely, without the clinician physically entering the room unless the physician determined closer assessment was warranted.

Infections associated with COVID-19 are expected to continue for the foreseeable future. As the country experiences spikes in hospitalizations, health systems will want to continue to be prudent about the use of PPE. Inpatient telemedicine provides a viable format for care delivery for some COVID-19 cases, as well as part of a general infection control strategy targeted at preventing the spread of other infectious conditions such as *Clostridium difficile* infection or multidrug-resistant organism infections causing sepsis.^9,10^ It is possible that hospital accreditation standards may incorporate the use of telemedicine as an approach to infection control.^9^ Experiences from early inpatient telemedicine adopters can provide lessons for the field as well as preliminary assessments of the impact on patient outcomes.

In this article, we describe University Hospital experience with launching their inpatient telemedicine program during the first 6 weeks of the COVID-19 pandemic. Specifically, we report on an analysis comparing transfers with the intensive care unit (ICU) or coronary care unit, use of ventilation, and length of ICU stay for individuals who received inpatient care through telemedicine relative to those who were not managed using telemedicine. In addition, we describe clinician experiences with inpatient telemedicine.

## Methods

### Data and measures

Data for this study are from adult UAB Hospital Medicine patients diagnosed with COVID-19 or suspected to have COVID-19 who were admitted to the two dedicated COVID-19 units between March 16, 2020 and April 24, 2020. Those units at that time were responsible for all hospitalized patients with a positive reverse transcription polymerase chain reaction, commonly known as reverse transcription polymerase chain reaction assay for COVID-19, and those suspected to have COVID-19. Those suspected to have COVID-19 were identified as any person with major symptoms suspicious for COVID-19, specifically fever, cough, and/or shortness of breath. Duplicate visits were omitted (i.e., if a patient was admitted twice within the time frame for COVID-19, only the initial visit was considered). The two COVID-19 units totaled 51 beds.

We conducted an analysis of clinical data obtained through chart abstraction from the University Hospital Enterprise Data Warehouse. Chart abstraction from University Hospital electronic medical records (EMRs) were performed by trained medical students supervised by clinician MDs and informaticians. Using patient medical record numbers, the students captured admission and discharge dates, confirmation of a COVID-19 diagnosis, demographic data, and whether the patient had hypertension, diabetes, and chronic pulmonary disease. This study was limited to these diseases because these are most associated with COVID-19 sequelae and are among University Hospital's highest comorbidities.^11,12^ Document analysis of the provider notes was used to indicate whether the patient received telehealth care and if so the number of telehealth visits.

This analysis examines the impact of telehealth use on three health outcomes: admission to the ICU, invasive ventilation after an inpatient telehealth encounter, and the number of census days in the ICU. We identified these critical COVID-19 outcomes as they have been adopted in other studies assessing the severity of infectious diseases, including COVID-19.^13–15^

The primary independent variable was receipt of any telehealth visits during the hospital stay, (1 = yes, 0 = no). Three sociodemographic patient characteristic variables were included in the analysis: age, gender, and race/ethnicity. Age was measured as a continuous variable and gender as a dichotomous variable (1 = female, 0 = male). Race/ethnicity was measured using three categories, black, white, and Asian/Latinx/other race/ethnicity, due to the low numbers of patients not identifying as black or white. The analysis also controlled for three clinical factors: presence of a cough (1 = yes, 0 = no), respiratory rate at admission, and the ratio of oxygen saturation level to delivered fraction of inspired oxygen (FiO_2_). Respiratory rate at admission was operationalized as a continuous variable measuring the number of respirations per minute when the patient was admitted to the service. The ratio of oxygen saturation level to delivered fraction of inspired oxygen (SpO_2_/FiO_2_) was used to measure the degree of acute respiratory distress syndrome in the patient.^16–18^ The oxygen saturation level was collected through pulse oximetry at admission. The FiO_2_ was approximated by the recorded supplemental oxygen delivery device and flow rate at admission, which was then converted to previously established corresponding FiO_2_ equivalents reported in the literature.^16–18^ Finally, the analysis accounted for three dichotomous indicators of comorbidity: hypertension, diabetes, and chronic pulmonary disease (1 = yes, 0 = no).

We employed a quasi-experimental design to compare outcomes for two different treatment models (telehealth or no telehealth) for inpatients with COVID-19 in the two University Hospital COVID-19 units. Patients who had telehealth encounters meant that they had agreed to use the tablet or telehealth chart. In addition, providers assessed a patient's ability to effectively communicate using the tablet or telehealth cart.

To gain perspectives of the clinical staff A.H. interviewed three hospitalists and the CCC on their experiences with implementation and interacting with patients using the inpatient telemedicine technologies for a total of four interviews. The interviews were conducted either by telephone or using an electronic video platform. The interview asked each interviewee to describe how telemedicine was implemented, how patients and other providers reacted to the technology, barriers and implementation facilitators, and the potential impact on health care quality. The interviews were taped and then transcribed. A.H. read the transcripts and identified key themes. Themes were then discussed with K.K., B.M., and K.M. (hospitalists on the COVID units) who clarified and confirmed themes. The interviews were intended to provide context and explanation for the quantitative findings and as such we used an inductive approach to generating themes.^19^

### Statistical analyses

We employed univariate and bivariate analyses to evaluate the relationship between receipt of telehealth and ICU admission, invasive ventilation, number of days in the ICU, and individual patient characteristics (comorbidities, clinical characteristics, and demographic characteristics) among those with COVID-19 seropositivity or suspected COVID-19 positive. We also conducted logistic regression modeling to estimate the statistical relationship between receipt of telehealth and ICU admission and invasive ventilation while controlling for the individual patient characteristics. Since the number of ICU days represents count data, we employed a negative binomial regression to estimate the relationship between ICU days and telehealth while controlling for individual patient characteristics. All models were estimated using Stata 16.1. (StataCorp. 2019. Stata Statistical Software: Release 16. College Station, TX: StataCorp LLC.)

### Ethical review and approval

The UAB Institutional Review Board deemed the study exempt from further review (IRB No. 300005213).

## Results

### Patient outcomes

Univariate and bivariate statistics are reported in Table 1. 6.67% of the patients received one telehealth visit (*n* = 9) and 17.04% received two or more telehealth visits (*n* = 23). Almost half of the sample were admitted to the ICU (48.89%, *n* = 66) and one-third received invasive ventilation (33.08%, *n* = 44). On average, if a patient was admitted to the ICU, they were in the ICU for 4.31 days, ranging from 1 to 28 days.

**Table 1.** Characteristics of the Sample (*n* = 135)

Patient characteristic	% of sample, *n* (%)	Number of telehealth visits			
		0 (*n* = 103), *n* (%)	1 (*n* = 9), *n* (%)	2+ (*n* = 23), *n* (%)	t/F/χ^2^
ICU/CCU admission					
Yes	66 (48.89)	46 (44.66)	8 (88.89)	12 (52.17)	6.60^*^
Ventilator					
Yes	44 (33.08)	32 (31.07)	4 (44.44)	8 (34.78)	0.735
Mean ICU census days (SD)	4.31 (6.38)	4.01 (6.22)	8.33 (9.38)	4.09 (5.41)	1.95
Sociodemographics					
Mean age (SD)	60.16 (16.48)	60.54 (16.45)	57.22 (13.82)	59.61 (18.04)	0.18
Gender					
Female	63 (46.67)	49 (47.57)	6 (66.67)	8 (34.78)	2.79
Male	72 (53.33)	54 (52.43)	3 (33.33)	15 (65.22)	
Race/ethnicity					
Black	73 (54.07)	55 (53.40)	7 (77.78)	11 (47.83)	2.78
Asian/Latinx/other	12 (8.90)	10 (9.71)	0 (0.00)	2 (8.70)	
White	50 (37.04)	38 (36.89)	2 (22.22)	10 (43.48)	
Clinical factors					
Cough					
Yes	60 (44.44)	46 (44.66)	3 (33.33)	11 (47.83)	0.56
Mean admission respiratory rate (SD)	19.74 (4.49)	19.68 (4.79)	21.22 (4.24)	19.43 (2.92)	6.99^*^
Mean pulse O_2_/Supplemental O_2_ (SD)	354.56 (123.47)	362.83 (122.57)	304.39 (117.21)	337.14 (128.60)	1.21
Comorbidities					
Hypertension					
Yes	94 (69.63)	71 (68.93)	8 (88.89)	15 (65.22)	1.81
Diabetes					
Yes	47 (34.81)	34 (33.33)	4 (44.44)	9 (39.13)	0.70
Chronic pulmonary disease					
Yes	25 (18.66)	18 (17.65)	3 (33.33)	4 (17.39)	1.37

Bold text refers to the statistically significant *p* values, ^*^p < 0.05.

CCU, coronary care unit; ICU, intensive care unit; SD, standard deviation.

We also examined relationships between the number of telehealth visits and sociodemographic characteristics, clinical factors, and comorbidities. The association between the number of telehealth visits and ICU admission showed statistical significance. About half of the patients with either no telehealth visits (44.66%, *n* = 46) or two telehealth visits (52.17%, *n* = 12) were admitted to the ICU, whereas almost 89% (*n* = 8) of the patients with one telehealth visit were admitted to the ICU (χ^2^ = 6.60, *p* < 0.05).

The average age in our sample was 60.16 years, ranging from 21 to 93 years, and 46.67% of the sample were female (*n* = 63). Half of the sample were black (54.07%, *n* = 73), 37.04% (*n* = 50) were white, and the remaining 8.90% (*n* = 12) were Asian, Latinx, or another race/ethnicity.

In terms of clinical factors and comorbidities, almost half of the sample presented with a cough (44.44%, *n* = 60), and the mean respiratory rate upon admission was 19.74 breaths per minute. The mean ratio of pulse O_2_ to supplemental O_2_ was 354.56. There were high levels of comorbidity in our sample. 69.63% of the sample also had a diagnosis of hypertension, 34.8% had diabetes, and 18.66% had chronic pulmonary disease.

There was also statistical significance in the difference in the mean respiratory rate by number of telehealth visits. Patients with no telehealth visits or two telehealth visits had, on average, 19.74 and 19.43 breaths per minute, respectively, whereas patients with one telehealth visit had, on average, 21.2 breaths per minute (*F* = 6.99, *p* < 0.05). There are no statistically significant differences between those who received telehealth and those who did not among sociodemographic characteristics, clinical factors, and comorbidity characteristics.

Logistic regression models were used to estimate the relationship between the receipt of inpatient telehealth, individual patient characteristics and the likelihood of ICU admission and ventilation. There were no statistically significant differences in the odds of admission to the ICU and the odds of ventilation between the patients who received telehealth and the patients who did not. The ratio of pulse O_2_ to supplemental O_2_ showed statistical significance in predicting admission to the ICU and invasive ventilation. As the ratio of pulse O_2_ to supplemental O_2_ increased, the odds of admission to the ICU and ventilation decreased (odds ratio [OR] 0.989, *p* < 0.001; OR 0.988, *p* < 0.001). Older ages were associated with higher odds of ICU admission (OR 1.037, *p* < 0.05). Increases in respiratory rate upon admission were associated with increased odds of ICU admission (OR 1.37, *p* < 0.001). Patients with a cough were associated with lower odds ventilation (0.287, *p* < 0.05).

Negative binomial regression models were used to estimate the relationship between telehealth, individual patient characteristics, and the number of days in the ICU. Patients presenting with a cough were associated with less days in the ICU (0.287, *p* < 0.05), and increases in the ratio of pulse O_2_ to supplemental O_2_ were associated with less days in the ICU (0.987, *p* < 0.001; Table 2).

**Table 2.** Correlates for Intensive Care Unit (ICU) Admission, Ventilator Use, and Number of Days in the ICU among Coronavirus Disease 2019 Patients Receiving Telehealth and Not Receiving Telehealth (*N* = 134)

	ICU admission	Ventilation	ICU census days
	OR (95% CI)	OR (95% CI)	IRR (95% CI)
Telehealth			
Yes	1.810 (0.603–5.438)	0.899 (0.294–2.749)	0.899 (0.294–2.749)
No	Referent	Referent	Referent
Sociodemographics			
Age	1.037 (1.006–1.069)^*^	1.001 (0.970–1.032)	1.001 (0.970–1.032)
Gender			
Female	1.056 (0.399–0.279)	1.039 (0.373–2.892)	1.039 (0.373–2.892)
Male	Referent	Referent	Referent
Race/ethnicity			
Black	1.796 (0.647–4.982)	1.388 (0.458–4.207)	1.388 (0.458–4.207)
Asian/Latinx/other	0.363 (0.051–2.562)	0.496 (0.047–5.289)	0.496 (0.465–5.289)
White	Referent	Referent	Referent
Clinical factors			
Cough			
Yes	0.469 (0.161–1.367)	0.287 (0.092–0.897)^*^	0.287 (0.092–0.897)^*^
No	Referent	Referent	Referent
Admission respiratory rate	1.337 (1.118–1.599)^**^	1.002 (0.893–1.124)	1.002 (0.893–1.124)
Pulse O_2_/supplemental O_2_	0.989 (0.985–0.995)^**^	0.988 (0.983–0.992)^**^	0.987 (0.983–0.992)^**^
Comorbidities			
Hypertension			
Yes	0.373 (0.106–1.311)	1.024 (0.307–3.409)	1.023 (0.307–3.409)
No	Referent	Referent	Referent
Diabetes			
Yes	2.579 (0.896–7.430)	1.255 (0.441–3.569)	1.255 (0.441–3.569)
No	Referent	Referent	Referent
Chronic pulmonary disease			
Yes	2.162 (0.641–7.286)	1.982 (0.646–12.087)	1.982 (0.559–7.028)
No	Referent	Referent	Referent

Bold text refers to the statistically significant *p* values, ^*^p < 0.05, ^**^p < 0.001.

CI, confidence interval; IRR, incident rate ratio; OR, odds ratio.

### Provider experiences with inpatient telehealth

Hospitalists and the CCC noted that inpatient telehealth interactions could be challenging given barriers such as confusion regarding how to use an electronic tablet, difficulty hearing the provider because of hearing impairments and the lack of headsets, underlying dementia or delirium, or the presence of a tracheotomy without a speaking valve. The CCC also noted that when she would call patients' rooms sometimes the patient would not answer, the patient could not locate the electronic tablet, the tablet was not within the patient's reach, the patient did not know the password to log on or how to log on, or the battery was depleted. Over time, the team recognized the importance of having a dedicated staff member focused on addressing technology issues and improving efficiency. Accordingly, the CCC's role evolved over time to focus exclusively on the use of the telemedicine technology.

Physicians noted that when inpatient telemedicine worked well, they were able to comfortably interact with the patient, see the patient's face, and the patient was able to see the provider's face without a mask. Physicians noted that this greatly enhanced interpersonal communication since each participant could read the other's expressions. Furthermore, physicians liked that efficient telemedicine interactions saved time by eliminating the 5–8 min typically used when donning and doffing PPE between in-person examinations and evaluations.

### Lessons learned

Physicians and the CCC noted two key lessons associated with their rapid implementation of telehealth in an inpatient setting. First, it is important to establish clear protocols for identifying those patients who were most appropriate for telehealth based on their mental status and ability to operate the technology. This required engaging with nursing to conduct and document mental assessments and to ensure this was documented in the EMR before in-person or virtual rounds. Although clinicians determined whether patients could engage in a telehealth visit, there was no systematic assessment of hearing, technological, or cognitive ability to provide an objective determination of suitability for a telehealth encounter. Second was a clear need to acknowledge the ethical issues associated with COVID-19 and that telemedicine should not be used in place of sound judgment about how to deliver appropriate medical care, regardless of the time or need for PPE. Because of the nature of academic medical centers and the expectation of trainees and students, the service needed to communicate clear guidelines that all patients, regardless of COVID status, must receive appropriate care. This included the expectation that physicians physically, in-person evaluate all patients upon admission as well as those patients who subsequently experience clinical deterioration, delirium, dementia, or any other condition that otherwise made them unsuitable for videoconferencing, especially where this was a change from baseline.

As the pandemic progressed throughout the summer months in 2020, University Hospital did not experience the surge in COVID-19 cases that was originally expected. In addition, the health system developed protocols for mask cleaning and gowns, and the need to preserve PPE was not as acute as anticipated. Furthermore, both patients and providers voiced preferences for in-person consultations. These factors in combination with technological barriers addressed earlier led to a gradual tapering of the use of inpatient telemedicine during physician rounds. However, nursing staff continue to use the technology to connect patients with their families or to facilitate clinical team–family member conferences. Since COVID-19 protocols at University Hospital prohibit family bedside visits, families especially appreciate being able to use telemedicine to see their loved ones.

### Limitations

This article reports on a small pilot study of a rapid implementation of an inpatient telehealth program in response to emerging concerns associated with the preservation of PPE during the early months of the COVID-19 pandemic. Accordingly, study limitations include the relatively short duration and the small number of patients included in the pilot. In addition, we had to employ a quasi-experimental design meaning we were not able to randomize patients to a telehealth or in-person care groups. More specifically, since the decision to participate in telehealth was based on the physician's assessment of the patient and the patient's willingness to participate, there is the possibility of selection bias. However, we do think we have utilized appropriate control variables in our analysis and reduced the potential for differences between the two groups.

## Discussion

This study reports findings from the use of telemedicine in confirmed and suspected COVID-19 positive patients. Although the initial purpose of implementing telemedicine was to conserve PPE, we quickly realized that was no longer an issue and focused our attention on the use of the technology on outcomes.

Bivariate analyses found that almost 89% (*n* = 8) of the patients with one telehealth visit were admitted to the ICU, compared with ∼50% of the patients with zero or two telehealth visits. It is important to note that there were some unique features of the patients receiving only one telehealth visit. A few of the patients were clinically unstable or decompensating quickly and the provider wanted to assess the patient in person, or the person was transferred quickly to the ICU. Some of the patients received one telehealth visit and expressed a preference to see their provider in person for the remainder of the hospitalization. Despite these initial differences in the bivariate analyses, statistically significant differences disappeared in the multivariate analyses once including sociodemographic and clinical control variables.

Loosely consistent with the literature, our analysis of a pilot demonstration of inpatient telehealth on a COVID-19 hospitalist unit found no statistically significant evidence of adverse outcomes among patients who received at least one telemedicine visit compared with those who had none^8^ and, therefore, may be appropriate as a means to infection control.^9^ Specifically, patients who received telehealth services were no more likely than patients who did not receive telehealth to be admitted to the ICU or be placed on a ventilator. Although not the primary focus of our study, we should note that that older individuals were more likely to be admitted to the ICU, as were individuals with higher respiratory rates upon admission. Individuals with a cough and lower ratios of pulse O_2_ to supplemental O_2_ were less likely to go to the ICU, to be placed on a ventilator, and to have fewer ICU days.

It is worth illuminating the broader implications of this study, even in light of its narrow focus within an academic medical center in the deep south. For example, our findings relative to the number of telehealth visits and ICU admissions may have implications on the frequency and timing of those telehealth visits. All too often technology implementations are deemed failures without the benefit of a root cause analysis—this can be accentuated in rapid implementations such as the requirement with COVID-19. Although it took us a while to realize the issue, our study findings highlight the need for having a dedicated staff member focused on implementation, end-user ability assessment, and overall use. This important finding provides guidance in broader patient-facing technology implementations.

It should be noted that the decision to communicate through telehealth in this study was based on provider judgment and patient willingness. These results suggest that telemedicine could be a safe and useful strategy in hospitalized patients with COVID-19, with a logical extension to connect patients in isolation with their families. However, we did note a set of implementation challenges including the need to assist patients who may not be technologically savvy. Regardless of the challenges, patients reported that they liked being able to see the physician unmasked. Findings from the project can inform continued development of telehealth modalities within the inpatient setting. Future inpatient telehealth projects should focus on making the technology more accessible and easier to use for both providers and patients. Although there was anecdotal reporting of time savings, more study is needed to quantify these time savings.

## Abbreviations Used

CCCs: clinical care coordinators
CI: confidence interval
COVID-19: coronavirus disease 2019
EMRs: electronic medical records
FiO_2_: fraction of inspired oxygen
ICU: intensive care unit
OR: odds ratio
PPE: personal protective equipment
SD: standard deviation
SpO_2_/FiO_2_: oxygen saturation level to delivered fraction of inspired oxygen
